# The case for prevention – Primary HIV prevention in the era of universal test and treat: A mathematical modeling study

**DOI:** 10.1016/j.eclinm.2022.101347

**Published:** 2022-03-14

**Authors:** Katharine Kripke, Robyn Eakle, Alison Cheng, Sangeeta Rana, Kristine Torjesen, John Stover

**Affiliations:** a Avenir Health, 6930 Carroll Ave., Suite 350, Takoma Park, MD 20912, USA; b U.S. Agency for International Development, Washington, DC, USA; c Department of Global Health and Development, London School of Hygiene and Tropical Medicine, London, UK; d FHI 360, Durham, NC, USA; e Avenir Health, Glastonbury, CT, USA

**Keywords:** HIV, Prevention, Mathematical modeling, Universal Test and Treat, Voluntary Medical Male Circumcision, Pre-exposure prophylaxis, Vaccine, Sub-Saharan Africa, Lesotho, Mozambique, Uganda, Goals model, Cost-effectiveness, test, testing

## Abstract

**Background:**

As antiretroviral therapy (ART) has scaled up and HIV incidence has declined, some have questioned the continued utility of HIV prevention. This study examines the role and cost-effectiveness of HIV prevention in the context of “universal test and treat” (UTT) in three sub-Saharan countries with generalized HIV epidemics.

**Methods:**

Scenarios were created in Spectrum/Goals models for Lesotho, Mozambique, and Uganda with various combinations of voluntary medical male circumcision (VMMC); pre-exposure prophylaxis; and a highly effective, durable, hypothetical vaccine layered onto three different ART scenarios. One ART scenario held coverage constant at 2008 levels to replicate prevention modeling studies that were conducted prior to UTT. One scenario assumed scale-up to the UNAIDS treatment goals of 90-90-90 by 2025 and 95-95-95 by 2030. An intermediate scenario held ART constant at 2019 coverage. HIV incidence was visualized over time, and cost per HIV infection averted was assessed over 5-, 15-, and 30-year time frames, with 3% annual discounting.

**Findings:**

Each prevention intervention reduced HIV incidence beyond what was achieved by ART scale-up alone to the 90-90-90/95-95-95 goals, with near-zero incidence achievable by combinations of interventions covering all segments of the population. Cost-effectiveness of HIV prevention may decrease as HIV incidence decreases, but one-time interventions like VMMC and a durable vaccine may remain cost-effective and even cost-saving as ART is scaled up.

**Interpretation:**

Primary HIV prevention is still needed in the era of UTT. Combination prevention is more impactful than a single, highly effective intervention. Broad population coverage of primary prevention, regardless of cost-effectiveness, will be required in generalized epidemic countries to eradicate HIV.

**Research in context**

**Evidence before this study**

The authors searched PubMed in October 2020 for studies dating from 2005 to 2020. Search terms included “mathematical modeling” OR “cost-effectiveness” AND “HIV” AND one of the following: “HIV prevention,” “voluntary medical male circumcision,” “pre-exposure prophylaxis,” and “vaccine.”

**Added value of this study**

Previous modeling has examined the impact of scaling up biomedical prevention in the context of expanded treatment or has attempted to present an optimized package of interventions, including expanded ART, but has not explored the role of prevention in the context of universal test and treat (UTT), nor examined the specific characteristics of prevention interventions necessary for impact and cost-effectiveness.

**Implications of all the available evidence**

Primary HIV prevention is still needed in the era of UTT. Preventing infections now with available interventions is more impactful and cost-effective than waiting for the perfect intervention. Combination prevention is more impactful than a single, highly effective intervention. In settings where intervention targeting to the highest risk populations has minimal impact, one-time interventions such as voluntary medical male circumcision provided to the general population are substantially more cost-effective than those that need to be delivered recurrently.

Alt-text: Unlabelled box

## Introduction

As antiretroviral therapy (ART) for HIV treatment has expanded to reach the UNAIDS 90-90-90/95-95-95 diagnosis, treatment, and viral suppression targets and evolved into universal test and treat (UTT),^1^ new HIV infections have declined, and the number of people living with HIV has increased.^2^ However, these numbers have stagnated in the last 2–3 years, according to global UNAIDS data.^2^ The impact of HIV treatment on slowing new infections and improving life expectancy among those infected has been definitively proven, however, the role of primary HIV prevention in this era has not yet been clearly defined.

Mathematical modeling has supported the case for new HIV prevention modalities, such as voluntary medical male circumcision (VMMC) and HIV vaccine development.^3^, ^4^, ^5^, ^6^ These studies demonstrated the impact and cost savings that could be achieved by introducing these new interventions, leading to massive rollout of VMMC programs in 15 priority countries in Southern and Eastern Africa. Modeling of oral pre-exposure prophylaxis (PrEP) largely concluded that cost-effectiveness would be achieved when PrEP is delivered to those at the highest risk or in settings where UTT has not been fully achieved.^7^, ^8^, ^9^, ^10^, ^11^, ^12^, ^13^ The WHO recommends that PrEP should be provided to populations at substantial risk of HIV, originally defined as those with incidence higher than 3% per year.^14^ Practically, however, few geographic areas with such high incidence have been identified, and behavioral risk scores have demonstrated poor prediction of HIV incidence,^15^ not to mention social harms and poor uptake associated with behavioral targeting. Even if precision targeting were possible, so few people would have access to PrEP, the resulting use would have little impact on epidemic control in the highest burden countries.^13^

Modeling has previously shown that HIV prevention is needed alongside ART to reach epidemic control^16^; however, cost-effectiveness analyses recommend high thresholds for investment. To date, newer prevention products have largely failed to reach the modeled levels of cost-effectiveness in the general population shown in the VMMC and vaccine modeling conducted prior to the expansion of UTT in resource-constrained settings.^4^^,^^5^^,^^17^ This has led to a number of questions. What is the role of primary prevention in the era of UTT? Why isn't HIV prevention always cost saving, like VMMC was in the initial analyses published in 2011? What drives cost-effectiveness, in addition to unit cost? Previous modeling has examined the impact of scaling up biomedical prevention in the context of expanded treatment,^18^ or has attempted to present an optimized package of interventions, including expanded ART,^19^ but has not explored the role of prevention in the context of UTT, nor examined the specific characteristics of prevention interventions necessary for impact and cost-effectiveness.

This paper seeks to explore responses to some of these questions using mathematical modeling of hypothetical scenarios in three sub-Saharan African countries. The scenarios presented here illustrate principles that can inform the rollout and further development of HIV prevention in the context of continued expansion and eventual maintenance of universal ART coverage.

## Methods

No ethics approval was sought for this mathematical modeling study, which used only data that was previously published and did not involve any human subjects.

Lesotho, Mozambique, and Uganda were chosen as examples of countries with generalized HIV epidemics, with different characteristics in terms of HIV incidence and ART scale-up trends.

### Models

The Goals and AIM models within the Spectrum suite of models have been described in detail elsewhere, including equations, parameter ranges and fitting procedures.^18^^,^^20^^,^^21^ Briefly, Spectrum is a suite of interacting dynamic, compartmental models, with an underlying demographic model. Spectrum/Goals (see Supplemental Figure 1) disaggregates the adult population ages 15–49 years into behavioral risk groups and estimates the impact and costs of scaling up HIV prevention and treatment interventions. The risk groups are defined as: (1) low-risk heterosexual (stable couples, defined as men and women reporting a single sexual partner in the last year); (2) medium-risk heterosexual (men and women with more than one partner in the last year); (3) high-risk heterosexual (female sex workers and their male clients); (4) men who have sex with men; and (5) male and female people who inject drugs. Spectrum is freely available, and the base Goals files are available upon request.

This set of analyses employs the Goals model calibrated to the nationally validated annual HIV prevalence and incidence estimates used by UNAIDS, produced within another module of Spectrum called AIM; the ART coverage and viral suppression rates also came from this source (Table 1). Behavioral data such as condom use, numbers of partners, and age of sexual debut are extracted from AIDS Indicator Surveys, Demographic and Health Surveys, and Population-based HIV Impact Assessment surveys.^22^, ^23^, ^24^, ^25^, ^26^, ^27^ HIV prevalence for each country model was fit to survey data as shown in Supplemental File 1. HIV incidence per 1000 in 2019 from the corresponding AIM file was 6.43 with an uncertainty interval of (5.44, 7.66) for Lesotho, 4.68 (2.9, 7.42) for Mozambique, and 1.38 (1.09, 1.87) for Uganda. Models were fit for each country by varying the values of key epidemiological parameters (probability of infection per contact; effects of factors affecting transmission per contact including primary stage infection, chronic stage infection, presence of other STIs, sex of susceptible partner, and viral suppression in infected partners) and comparing the estimated prevalence and incidence with survey and surveillance data, including their confidence intervals. Output uncertainty intervals around HIV prevalence and incidence are 95% plausibility bounds resulting from selecting alternative parameter combinations according to their goodness of fit to the data.

**Table 1:** ART and VMMC coverage (percent of the adult HIV+ population and percent of the adult male population, respectively) and viral suppression levels (percent of those on ART) from the Goals files calibrated to the 2020 AIM files for each country.

Country	Lesotho	Mozambique	Uganda
Male ART coverage in 2008	18	9	16
Female ART coverage in 2008	18	11	14
Viral suppression in 2008	94	78	91
Male ART coverage in 2019	57	48	77
Female ART coverage in 2019	69	65	88
Viral suppression in 2019	94	78	91
VMMC coverage in 2008	19	51	25
VMMC coverage in 2019	49	71	65

### ART scenarios

Three ART coverage scenarios were created for this analysis (Table 2). The “2008” scenario was created to have similar assumptions to those used in earlier VMMC modeling.^4^ In the “2008” scenario, ART and VMMC coverage were held constant at 2008 levels through 2050. In the “2019” scenario, ART coverage was held constant at 2019 levels through 2050, to represent what might happen if countries failed to achieve 95-95-95 targets. At the time this analysis was conducted, 2019 was the most recent year of validated program data for estimating ART coverage. In the “95-95-95” scenario, ART coverage was scaled up from 2019 levels to 81% in 2025 (representing 90% of HIV-positive people knowing their status and 90% of those people being on ART) and 90% by 2030 (representing 95% of HIV-positive people knowing their status and 95% of those people being on ART). All scale-up in this paper used linear interpolation between the indicated values. Viral suppression on ART was scaled from 2019 levels to 90% in 2025 and 95% in 2030. For countries that had already reached or exceeded the first set of targets by 2019, the 2025 intermediate scale-up was skipped, and the levels were scaled directly from 2019 levels to the 95-95-95 targets by 2030. VMMC levels for the “2019” and “95-95-95” scenarios remained as they were in the originally calibrated file through 2019 (reflecting actual scale-up of VMMC between 2008 and 2019) and were then held constant at 2019 levels unless otherwise indicated.

**Table 2:** ART coverage and viral suppression levels for each scenario.

ART scenario	Parameter	Initial value	Initial year	Value in 2025	Value in 2030	Value in 2050
2008	ART coverage	2008 levels	2008	2008 levels	2008 levels	2008 levels
2008	Viral suppression	2008 levels	2008	2008 levels	2008 levels	2008 levels
2019	ART coverage	2019 levels	2019	2019 levels	2019 levels	2019 levels
2019	Viral suppression	2019 levels	2019	2019 levels	2019 levels	2019 levels
95-95-95	ART coverage	2019 levels	2019	81%	90%	90%
95-95-95	Viral suppression	2019 levels	2019	90%	95%	95%

### Primary prevention scale-up scenarios

A selection of scenarios was developed for scaling up VMMC, oral PrEP, and a hypothetical HIV vaccine, then layered individually and in combination with the three ART scenarios. Table 3 shows the scale-up patterns for each prevention intervention. After scale-up, coverage of each prevention intervention was maintained at the indicated scale-up coverage value through 2050. VMMC was assumed to provide a 60% (51%–64%) reduction in HIV incidence among heterosexual adult males and was applied among males ages 15–49 years.^28^, ^29^, ^30^ Oral PrEP was assumed to have 95% efficacy and 75% adherence,^31^^,^^32^ resulting in an overall effectiveness of 71% (61%–81%).^33^ Coverage levels for oral PrEP indicated in Table 3 were among medium- and high-risk heterosexual men and women, except for the “PrEP All” scenarios, in which coverage extended to all populations represented in the Goals model. Unlike HIV vaccines tested to date, for the purpose of this analysis, the HIV vaccine was optimistically assumed to have 80% efficacy (reduction in acquisition) and a 30-year duration of protection. The vaccine was scaled up among all adults.

**Table 3:** Scale-up patterns for HIV prevention interventions.

ART scenario	Prevention intervention	Initial coverage value	Initial year	Scale-up coverage value	Scale-up target year
2008	Oral PrEP	0%	2011	30%	2016
2019	Oral PrEP	0%	2019	30%	2024
95-95-95	Oral PrEP	0%	2019	30%	2024
2008	PrEP All	0%	2011	80%	2016
2019	PrEP All	0%	2019	80%	2024
95-95-95	PrEP All	0%	2019	80%	2024
2008	VMMC	2008 level	2011	80%	2016
2019	VMMC	2019 level	2019	80%	2024
95-95-95	VMMC	2019 level	2019	80%	2024
2008	HIV vaccine	0%	2020	80%	2025
2019	HIV vaccine	0%	2030	80%	2035
95-95-95	HIV vaccine	0%	2030	80%	2035

The combination of prevention and ART scenarios is depicted in Table 4. To calculate HIV infections averted and incremental costs, each prevention scale-up scenario was compared with the corresponding ART base scenario without any prevention scale-up. HIV infections averted and incremental costs were counted for 5, 15, and 30 years starting in 2012 for the 2008 scenarios and 2020 for the 2019 and 95-95-95 scenarios.

**Table 4:** Scenarios generated for each of the three countries.

Scenario name	ART	PrEP	PrEP-all	VMMC	Vaccine
2008	2008				
2008_PrEP	2008	X			
2008_PrEPAll	2008		X		
2008_VMMC	2008			X	
2008_vaccine	2008				X
2008_PrEP_VMMC	2008	X		X	
2008_PrEP_VMMC_vaccine	2008	X		X	X
2019	2019				
2019_PrEP	2019	X			
2019_PrEPAll	2019		X		
2019_VMMC	2019			X	
2019_vaccine	2019				X
2019_PrEP_VMMC	2019	X		X	
2019_PrEP_VMMC_vaccine	2019	X		X	X
95_95_95	95-95-95				
95_95_95_PrEP	95-95-95	X			
95_95_95_PrEPAll	95-95-95		X		
95_95_95_VMMC	95-95-95			X	
95_95_95_vaccine	95-95-95				X
95_95_95_PrEP_VMMC	95-95-95	X		X	
95_95_95_PrEP_VMMC_vaccine	95-95-95	X		X	X

### Costs

Fully loaded, provider side delivery costs of ART, VMMC, PrEP, and the vaccine were included in this analysis. All costs were converted to 2019 USD using Gross Domestic Product deflator values from the U.S. Department of Commerce Bureau of Economic Analysis website.^34^ The unit costs and sources for each intervention for each country are listed in Table 5. The PrEP-it Cost Lite module,^35^ which provides cost estimates based on an analysis of primary data from six countries, was used to estimate the per person cost for a full year of PrEP for each of the three countries. Default personnel costs available in the tool were used for each country. Lab costs from the PrEP Cost Model South Africa were available in the Cost Support tab of the tool.^36^ The South Africa costs were converted from Rand to USD using the conversion rate for July 1, 2018 (mid-year) from www.xe.com, accessed November 3, 2020. Costs were averaged across the four populations listed in the tool. Other recurrent and capital costs were calculated from the default ratios available in the tool. Average per person per year antiretroviral drug costs (USD, 2017) for emtricitabine 200 mg + tenofovir 300 mg tabs for the Eastern and Southern Africa region were obtained from the UNAIDS HIV Financial Dashboard.^37^ Both costs and HIV infections averted were discounted at 3% per year to facilitate comparison with similar analyses.

**Table 5:** Unit costs used in the analysis, and their sources. pppy = per person per year.

Country	Intervention	Unit Cost (USD)	Units	Source
Lesotho	ART	$113·94	pppy	Nichols et al.^38^
Mozambique	ART	$234·51	pppy	Korenromp et al.^39^
Uganda	ART	$154·34	pppy	Do DSD Models for HIV Treatment Save Money for Health Systems? (presentation)^40^
Lesotho	VMMC	$68·45	per circumcision	Unit Cost Study Repository^41^
Mozambique	VMMC	$39·90	per circumcision	Korenromp et al.^39^
Uganda	VMMC	$49·54	per circumcision	Unit Cost Study Repository^41^
Lesotho	PrEP	$123·06	pppy	PrEP-it Cost Lite module.^35^ See text for details.
Mozambique	PrEP	$118·45	pppy	PrEP-it Cost Lite module.^35^ See text for details.
Uganda	PrEP	$111·85	pppy	PrEP-it Cost Lite module.^35^ See text for details.
Lesotho	HIV vaccine	$26·95	per full course of vaccination	Moodley et al.^42^ Used the high end of the range of costs used in the study.
Mozambique	HIV vaccine	$26·95	per full course of vaccination	Moodley et al.^42^ Used the high end of the range of costs used in the study.
Uganda	HIV vaccine	$26·95	per full course of vaccination	Moodley et al.^42^ Used the high end of the range of costs used in the study.

### Cost sensitivity analysis

Intervention unit costs can vary substantially, depending on service delivery model, demand, implementation efficiencies, fluctuating commodity costs, and other factors. For the cost-effectiveness analysis, we varied the unit costs of each intervention – ART, VMMC, PrEP, and the hypothetical HIV vaccine – between 50% and 150% of the base costs listed in Table 5.^43^^,^^44^ For each scenario reported, in addition to the cost per HIV infection averted using the base costs, we reported the minimum and maximum cost per HIV infection averted across the cost sensitivity analyses.

### Role of the funding source

Study sponsors played no role in the design or conduct of the analysis. All authors had access to the data and took the decision to submit the paper for publication.

## Results

### What is the role of primary prevention in the era of UTT?

**Figure 1:**
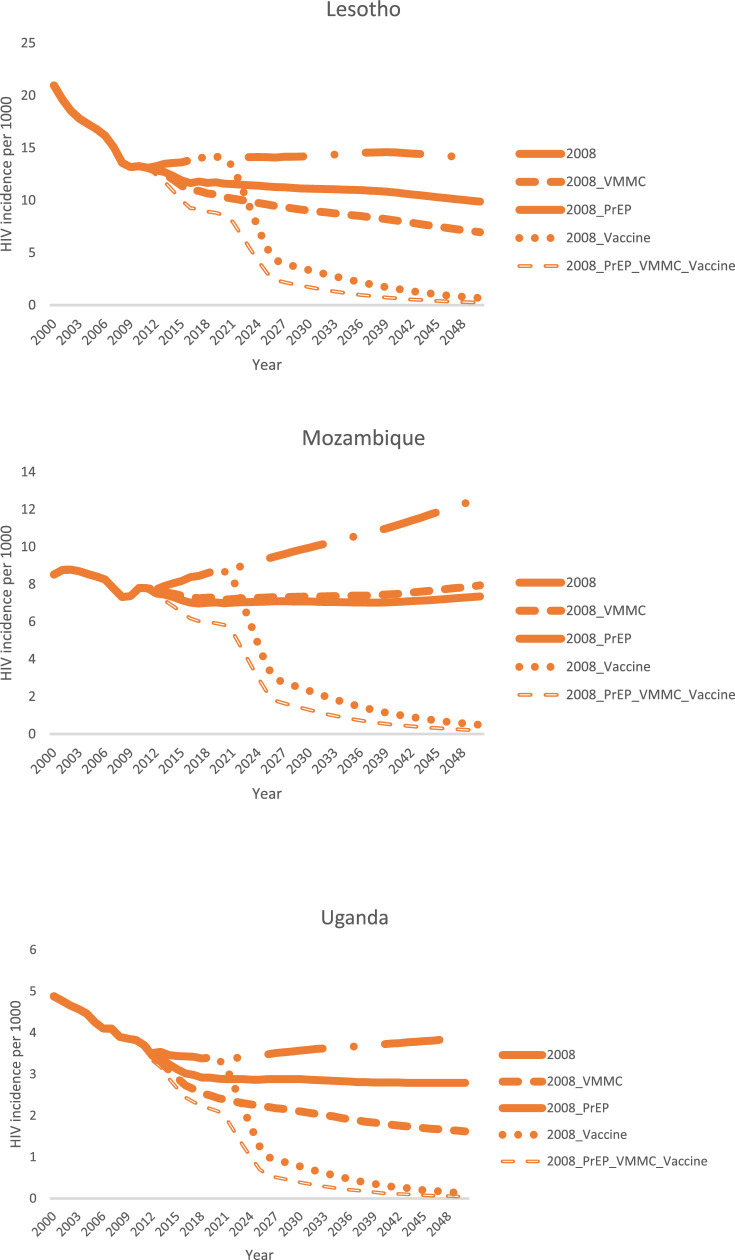
Modeled reduction in total population (all ages, male and female) HIV incidence from scaling up VMMC, PrEP, an HIV vaccine, and a combination of all three, when ART coverage is held constant at 2008 levels. Scenario names are explained in Table 4.

Figure 1 shows how VMMC, oral PrEP, and a hypothetical highly effective HIV vaccine would have been projected to decrease HIV incidence when ART coverage was held constant at 2008 coverage levels in Lesotho, Mozambique, and Uganda. This set of scenarios is similar to those used in the early VMMC and vaccine modeling exercises. In all three countries, HIV incidence was substantially impacted by each of the interventions, with the impact increasing in line with the assumed effectiveness of the intervention. VMMC scaled up to 80% coverage had higher impact than oral PrEP scaled up to 30% coverage, except in Mozambique, where the baseline male circumcision prevalence was substantially higher, and therefore the increase in male circumcision coverage when scaling up to 80% was less, compared with the other two countries. If both VMMC and PrEP were scaled up to 80% coverage, VMMC, with an assumed efficacy of 60%, had a lower impact than oral PrEP, with an assumed effectiveness of 71% (Supplemental Figure 2). The HIV vaccine, with assumed efficacy of 80% and scaled up to 80% coverage, had the greatest impact of any of the individual interventions. Combining all three interventions brought HIV incidence down to nearly zero by 2050 in all three countries.

**Figure 2:**
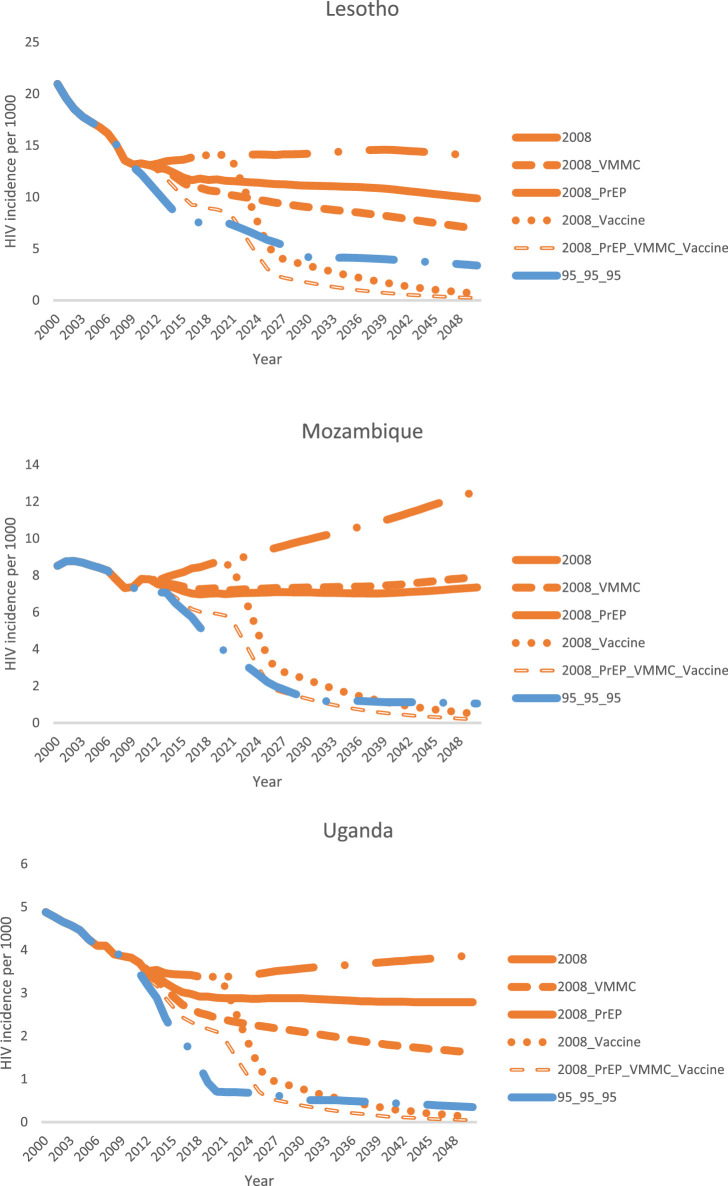
Comparison of modeled total population (all ages, male and female) HIV incidence reduction from scaling up ART to reach the Fast-Track targets and VMMC to 2019 levels with what could have been achieved by scaling up VMMC, PrEP, an HIV vaccine, or a combination of all three, if ART coverage were held constant at 2008 levels. Scenario names are explained in Table 4.

In Figure 2, we added a scenario (“95-95-95”) in which ART coverage was scaled up from 2019 levels to 90-90-90 targets by 2025 and to 95-95-95 targets by 2030. In this scenario, VMMC and ART coverage followed actual country scale-up trends between 2008 and 2019, while oral PrEP and an HIV vaccine were not introduced. This figure demonstrates that scaling up ART according to the 95-95-95 targets, along with actual VMMC scale-up between 2008 and 2019 followed by maintenance of VMMC at 2019 coverage levels, can decrease HIV incidence to levels comparable to what could have been achieved by scaling up an 80% effective HIV vaccine by 2025 while holding ART and VMMC constant at 2008 levels.

**Figure 3:**
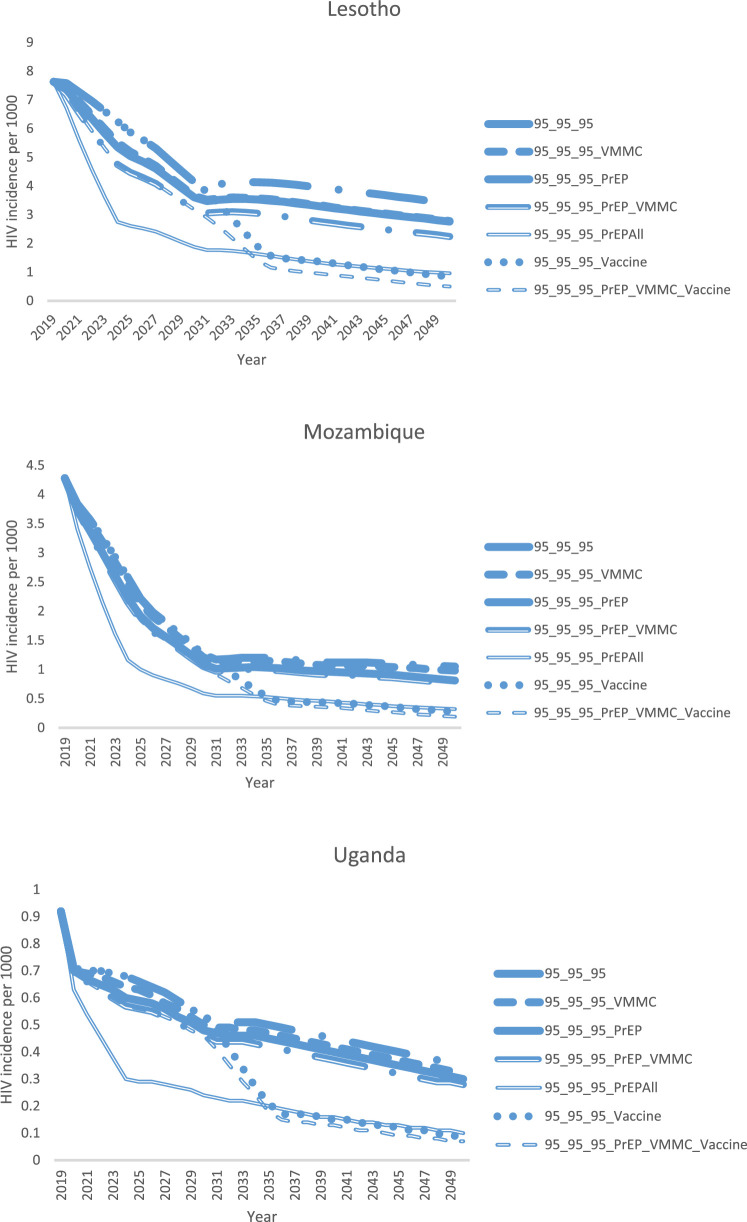
Modeled reduction in total population (all ages, male and female) HIV incidence from scaling up VMMC, PrEP, an HIV vaccine, both VMMC and PrEP, and a combination of all three, when ART coverage is scaled up to the 90-90-90/95-95-95 targets. Scenario names are explained in Table 4.

Figure 3 explores the additional projected impact attained by scaling up primary HIV prevention in the context of achieving the 90-90-90/95-95-95 targets for ART. Lesotho provides the most dramatic example, but the trends are similar in all three countries. Firstly, a highly effective vaccine, scaled up to 80% coverage of the general population by 2030, could reduce HIV incidence by an additional 70–75% by 2050, compared with the incidence in the 95-95-95 base scenario. Secondly, the combination of oral PrEP and VMMC could reduce HIV incidence by an additional 20% in Uganda, 22% in Mozambique, and 34% in Lesotho, compared with the 95-95-95 scenario. VMMC scale-up in the 95-95-95 scenarios was projected to have less of an impact in Mozambique and Uganda, since these countries are estimated to have already achieved relatively high male circumcision coverage by the end of 2019. Thirdly, the “PrEP All” scenario, in which oral PrEP is scaled up to 80% coverage of all populations, achieved the same HIV incidence reduction as the HIV vaccine scale-up scenario. This demonstrates that what is important for incidence reduction is not the specific platform (PrEP or vaccine), but rather how broadly within the population coverage can be achieved, along with the effectiveness of the intervention. And finally, the greatest impact was projected to be achieved by the PrEP_VMMC_Vaccine scenario, demonstrating that a combination of different prevention options for different population segments is ultimately the most impactful strategy for ending the HIV epidemic.

### Sensitivity and uncertainty analyses

**Figure 4:**
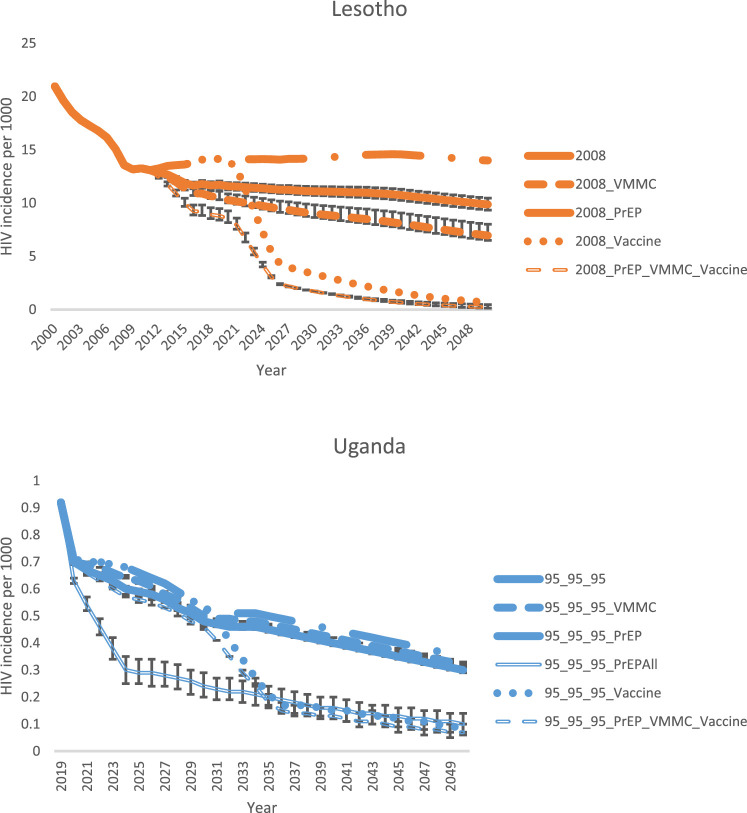
Uncertainty around modeled reduction in total population (all ages, male and female) HIV incidence from scaling up VMMC, PrEP, and a combination of VMMC, PrEP, and a vaccine, based on uncertainty in VMMC and PrEP effectiveness. The first panel shows Lesotho when ART coverage is held constant at 2008 levels (highest HIV incidence scenario, and the second panel shows Uganda when ART coverage is scaled up to the 90-90-90/95-95-95 targets (lowest HIV incidence scenario). Scenario names are explained in Table 4.

To demonstrate the impact of different coverage levels, Supplemental Figure 3 shows the impact if PrEP were scaled up to 80% coverage in the PrEP, PrEP_VMMC, and PrEP_VMMC_Vaccine scenarios. Figure 4 demonstrates the impact of uncertainty around VMMC (51–64%) and PrEP (61–81%) effectiveness, using Lesotho in the 2008 scenario (with the highest overall HIV incidence), and Uganda in the 95-95-95 scenario (with the lowest overall HIV incidence) as examples. Supplemental Figure 4 shows the magnitude of the uncertainty around the incidence estimate itself.

### What if we do not reach 95-95-95?

**Figure 5:**
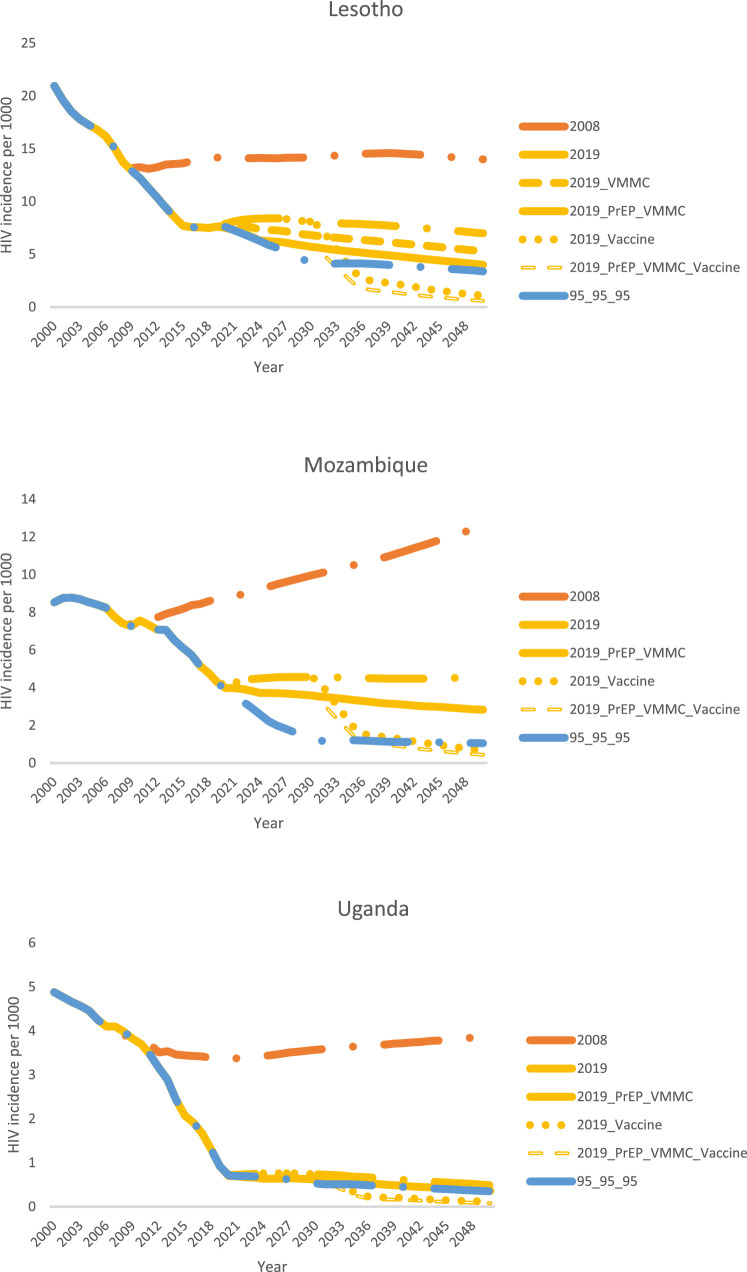
Modeled reduction in total population (all ages, male and female) HIV incidence from scaling up combinations of VMMC, PrEP, and an HIV vaccine, when ART coverage is maintained constant at 2019 levels. The scenario of holding ART coverage constant at 2008 levels and the scenario of scaling up ART to reach the 90-90-90/95-95-95 targets without primary prevention scale-up are included as comparators. Scenario names are explained in Table 4.

The scenarios presented above assume that the 90-90-90 and 95-95-95 ART targets can be met and maintained. However, some countries are struggling to attain these goals, and disruptions such as the COVID-19 pandemic can cause treatment interruptions and delays in scaling up. Figure 5 shows that if the 95-95-95 targets are not met, primary prevention becomes even more important for attaining epidemic control. In Lesotho, continuing to scale up VMMC can mitigate increases in HIV incidence that would occur if ART were not scaled up beyond 2019 levels. In Lesotho and Uganda, if ART coverage is maintained constant at 2019 levels, scaling up both VMMC for men and oral PrEP for medium- and high-risk men and women can bring HIV incidence down close to what could be achieved from scaling up ART to 95-95-95 without further primary prevention scale-up, but in Mozambique, higher coverage of primary prevention would be needed to achieve the same HIV incidence reduction as scaling up ART to 95-95-95. As was demonstrated in the other two ART scenarios, adding a highly effective vaccine for the general population, either alone or in combination with PrEP and VMMC scale-up, can provide further reductions in HIV incidence in all three countries.

### What drives cost-effectiveness of HIV prevention?

As shown in Table 6, in all countries, across all time frames, one-time interventions (VMMC and the hypothetical highly effective, long-acting HIV vaccine) are substantially more cost-effective than oral PrEP, which needs to be delivered on a recurrent basis throughout an individual's period of risk. In all but two of the scenarios (Lesotho 2008 5-yr and Mozambique 95-95-95 15-yr), the highest cost per HIV infection averted (HIA) from the cost sensitivity analysis for both VMMC and the vaccine is lower than the lowest cost per HIA for oral PrEP. In many of the settings and time frames explored in this analysis, VMMC and the HIV vaccine are cost-saving (negative cost per HIV infection averted), even in the context of 95-95-95. This trend is even more pronounced when considering the minimum cost per HIA from the cost sensitivity analyses. As HIV incidence comes down between the 2008 scenario and the 2019 and 95-95-95 scenarios, the cost per HIV infection averted goes up for VMMC and oral PrEP, at least in the short term (five-year time frame). In the medium and long term, this trend continues for oral PrEP. However, there is no clear trend in the relationship between cost-effectiveness and the ART scenario in the 15- and 30-year time frames for VMMC and the vaccine, as there is a tradeoff between greater HIV incidence reduction in the 2008 scenario and greater treatment cost savings in the 2019 and 95-95-95 scenarios with dramatically increased ART coverage. Finally, for VMMC and the HIV vaccine, the cost per HIV infection averted goes down across all scenarios as the time frame increases from five to 15 to 30 years.

**Table 6:** Cost per HIV infection averted for VMMC, oral PrEP, and a long-acting, highly effective HIV vaccine, under three different ART coverage scenarios, across three time frames. Minimum and maximum from cost sensitivity analysis are in parentheses. Negative numbers indicate that the scenario is cost saving compared with the counterfactual of not scaling up prevention.

Country	Scenario	Prevention Intervention	Cost/HIA 5 yr	Cost/HIA 15 yr	Cost/HIA 30 yr
Lesotho	2008	VMMC	1819 (907, 2731)	427 (158, 695)	106 (-120, 333)
		PrEP	4732 (2363, 7101)	4426 (2152, 6700)	4062 (1843, 6281)
		Vaccine	N/A	555 (274, 836)	92 (-54, 239)
	2019	VMMC	2552 (1193, 3912)	382 (-181, 945)	-241 (-810, 327)
		PrEP	9105 (4461, 13,748)	8973 (4083, 13,863)	8196 (3360, 13,032)
		Vaccine	N/A	1653 (766, 2540)	-86 (-539, 368)
	95-95-95	VMMC	2655 (1235, 4075)	695 (-117, 1506)	-51 (-848, 746)
		PrEP	9330 (4571, 14,089)	13,201 (6129, 20,274)	14,003 (6160, 21,847)
		Vaccine	N/A	2779 (1323, 4235)	171 (-464, 807)
Mozambique	2008	VMMC	1073 (534, 1612)	190 (39, 342)	-45 (-186, 97)
		PrEP	4285 (2140, 6430)	3331 (1600, 5062)	2416 (1033, 3798)
		Vaccine	N/A	607 (299, 915)	88 (-68, 243)
	2019	VMMC	1902 (811, 2992)	-87 (-644, 471)	-862 (-1555, -169)
		PrEP	9746 (4714, 14,779)	8170 (3405, 12,934)	6131 (1842, 10,421)
		Vaccine	N/A	1947 (859, 3035)	-395 (-1096, 307)
	95-95-95	VMMC	2334 (982, 3685)	322 (-823, 1468)	-738 (-2108, 632)
		PrEP	11,743 (5683, 17,802)	19,811 (8932, 30,689)	24,333 (10,434, 38,232)
		Vaccine	N/A	6636 (3180, 10,091)	612 (-882, 2106)
Uganda	2008	VMMC	3601 (1798, 5405)	822 (361, 1284)	303 (20, 587)
		PrEP	13,445 (6720, 20,170)	11,396 (5644, 17,148)	9083 (4406, 13,760)
		Vaccine	N/A	1615 (804, 2426)	449 (146, 753)
	2019	VMMC	16,722 (8218, 25,227)	4385 (1579, 7192)	1840 (-242, 3922)
		PrEP	71,681 (35,690, 107,672)	73,433 (36,064, 110,802)	70,562 (34,061, 107,064)
		Vaccine	N/A	13,334 (6559, 20,108)	2878 (574, 5182)
	95-95-95	VMMC	14,938 (7344, 22,531)	5103 (1921, 8285)	2705 (181, 5229)
		PrEP	63,629 (31,685, 95,573)	80,635 (39,688, 121,583)	87,206 (42,426, 131,986)
		Vaccine	N/A	15,228 (7521, 22,935)	3496 (955, 6037)

## Discussion

This modeling analysis demonstrates that primary prevention is still needed to reduce HIV incidence beyond what is achievable with ART scale-up, even in the context of significant ART contributions to decreased community transmission of HIV through progress toward the 95-95-95 targets. This is particularly important in countries that have been slow to scale up ART, as demonstrated in the 2019 scenario for Lesotho and Mozambique. Importantly, it is possible for primary prevention to decrease HIV incidence by an additional 70–75% compared with reductions that could be achieved by scaling up ART alone, particularly with highly efficacious prevention interventions delivered broadly to the general population at high coverage levels. In addition, it showed that combinations of prevention interventions with broad population coverage provide the greatest impact.

This analysis demonstrates that for one-time interventions, the cost per HIV infection averted decreases (cost-effectiveness increases) as the time horizon of analysis increases. Therefore, they are likely to remain cost-effective, or even cost-saving when the cost of the prevention is less than the cost of treatment over time. As incidence declines, higher cost interventions that need to be delivered on a recurrent basis, like PrEP, would only be cost-effective in individuals during phases of highest risk or in localities with high incidence. The size of the population in which PrEP would be considered cost-effective will shrink as overall incidence drops.

These findings provide important context for decision-makers and funders that are considering supporting novel HIV prevention interventions and planning for the future of large-scale HIV prevention and treatment programs. This analysis shows that cost-effectiveness findings from today cannot be compared with those published ten years ago, given significant changes in the epidemic where ART has expanded and HIV incidence has declined. Rather, it is critical to consider impact and cost-effectiveness of new HIV prevention interventions, and how they influence decision-making, differently than in the past. For instance, based on cost-effectiveness modeling, the trend among funders in recent years has focused narrowly on HIV prevention among populations and geographies with the highest HIV incidence, resulting in limited reach and impact of interventions. While more expensive than other prevention modalities, oral PrEP has shown dramatic decreases in new infections, even controlling for contributions from ART, regardless of type of epidemic and imperfect adherence.^45^^,^^46^ In addition, preventing an HIV infection today, even with an intervention that is relatively less cost-effective than other currently available interventions, is more cost-effective than preventing an HIV infection in the future, as prevention today will lead to fewer downstream infections in the future, as well as lower ART costs. With this in mind, if using theoretical cost-effectiveness thresholds as the only criterion for resourcing prevention, the field will never end HIV transmission in countries with generalized epidemics, even if the 90-90-90 and 95-95-95 targets are achieved. For countries in which a large proportion of HIV transmission is occurring among people who are not easily identifiable as “high risk,” primary HIV prevention will need to be available generally, now, and with high coverage, to have a substantial, sustained impact on HIV incidence. Prevention provided to people with lower risk will be less cost-effective, but it may be necessary to achieve population-level impact.

A broadly available, comprehensive package of prevention options will provide the greatest impact, as roughly demonstrated in our scenarios combining VMMC, PrEP, and the hypothetical vaccine. For men and women this could include a method mix of condoms and lubricants, VMMC, varieties of PrEP (oral, topical, injectable), and eventually a vaccine. Even with a highly effective, long-acting vaccine available, additional impacts can be gained by providing multiple prevention interventions to different segments of the population, providing support for the concepts of choice and combination prevention.

Cost-effectiveness may vary for new biomedical prevention products depending on the product and mode of delivery. However, the cost of maintaining epidemic control, and even reaching eradication, must be considered. Even though the cost to prevent an HIV infection by any intervention may increase as HIV incidence decreases, discontinuing HIV prevention in the context of epidemic control would likely bring substantial risk for resurgence as people move around and contextual risks shift, potentially regressing to endemic levels of HIV in a given country.

This analysis highlights an important point for consideration as new HIV vaccine candidates are advanced in the pipeline: whether and how frequently they require periodic boosting. Recent trial results of long-acting injectable cabotegravir foreshadow the possibility of PrEP delivery modalities that require less frequent administration, where a vaccine that needs continual boosting could be competing with long-acting PrEP. To be a game-changer compared to PrEP, a vaccine would need at least two of the following characteristics: (1) administered less frequently than the longest-acting PrEP delivery technology; (2) at least as effective as PrEP; (3) side effect, cost, and supply chain profile such that it can easily and affordably be provided to the general population.

As with any modeling study, the specific HIV incidence and cost-effectiveness values presented here are a function of the input data, which carry inherent uncertainties. Projections into the future are particularly uncertain, as both future HIV incidence and future costs depend on numerous factors that are impossible to predict. The cost-effectiveness values are sensitive to the relative costs of the various prevention interventions and ART. Since the costs of these interventions are not all derived from the same study, the methodology of determining the unit costs of the different interventions in different countries may not be comparable. Oral PrEP costs may go down as implementation efficiencies are identified, and its cost-effectiveness will also increase if, as was demonstrated in the SEARCH and other studies,^31^^,^^47^, ^48^, ^49^ users correctly identify periods when they are at higher risk and use the intervention during these periods.^50^ Despite these limitations, the overall trends identified in this study of three sub-Saharan African countries with different epidemic characteristics should be applicable to other sub-Saharan African countries with generalized HIV epidemics. Some of the conclusions, most notably the importance of providing primary prevention to people who are not “high risk,” will not be applicable in settings with concentrated HIV epidemics.

Primary HIV prevention is still needed in the era of UTT. Preventing infections now is more impactful and cost-effective than waiting for the perfect intervention. Combination prevention is more impactful than a single, highly effective intervention. While it will be less cost-effective, primary prevention must be provided to lower risk populations in generalized epidemic countries for prevention to have an epidemiological impact. In these settings, one-time interventions such as VMMC are substantially more cost-effective than those that need to be delivered recurrently. Finally, the field will need to take treatment cost savings into account as it grapples with the cost of maintaining epidemic control and future eradication of HIV.

## Declaration of interests

KK and JS are employed by Avenir Health and conducted this work under the above-cited grants from USAID through a subaward from FHI 360.

RE, AC, and SR are employed by USAID.

KT is employed by FHI 360 and contributed to this work under the above-cited grants from USAID.

## Appendix Supplementary materials

Image, application 1

Image, application 2

Image, application 3

Image, application 4

Image, application 5
