# Supplementary figures and images for: The case for prevention – Primary HIV prevention in the era of universal test and treat: A mathematical modeling study
Source: eClinicalMedicine. 2022 Mar 14;46:101347. doi: 10.1016/j.eclinm.2022.101347 (PMC8924323; doi:10.1016/j.eclinm.2022.101347)

# Goals Model

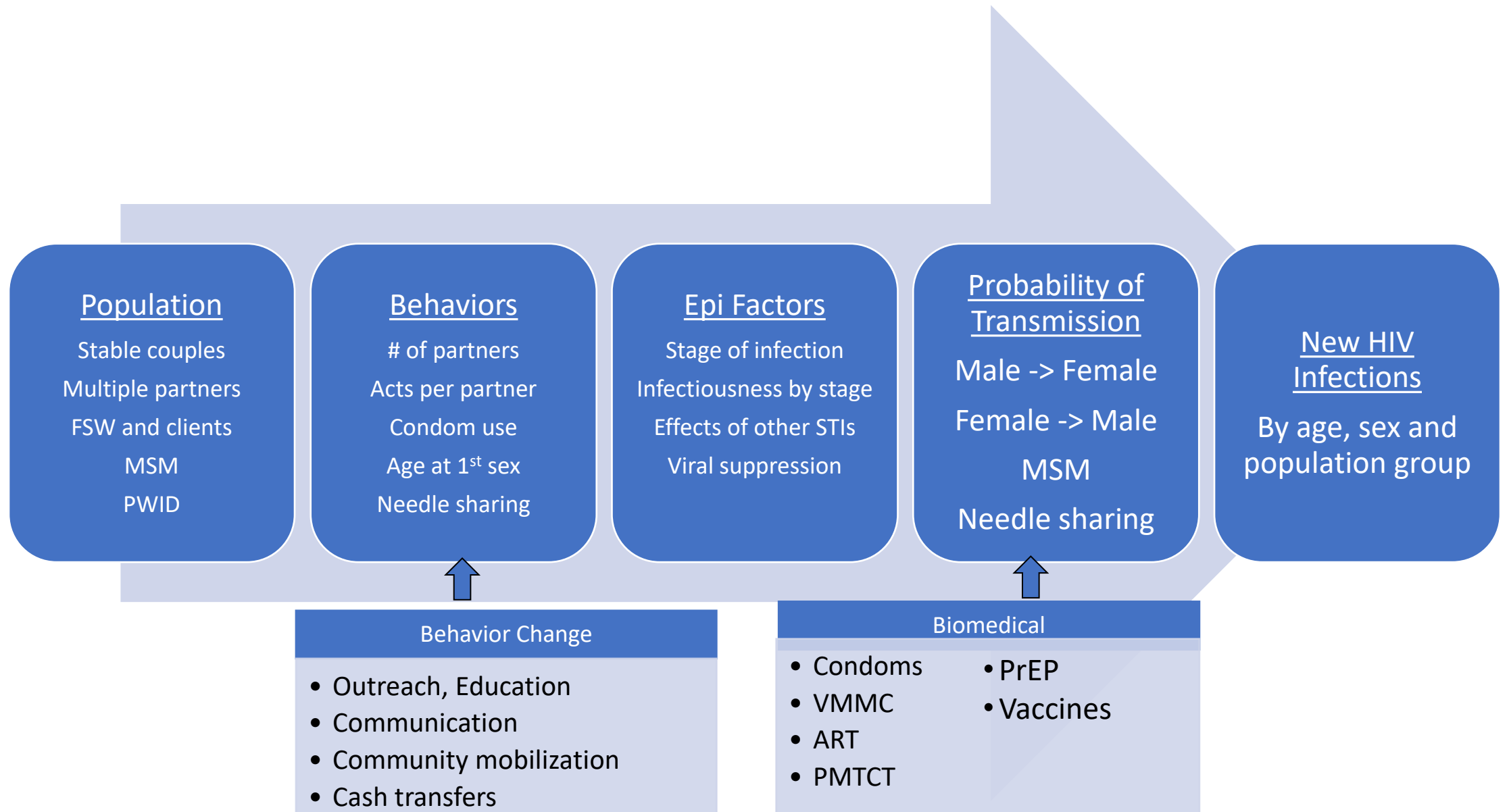

Supplement: Supplementary file 1 [file mmc1.pdf]

# Lesotho

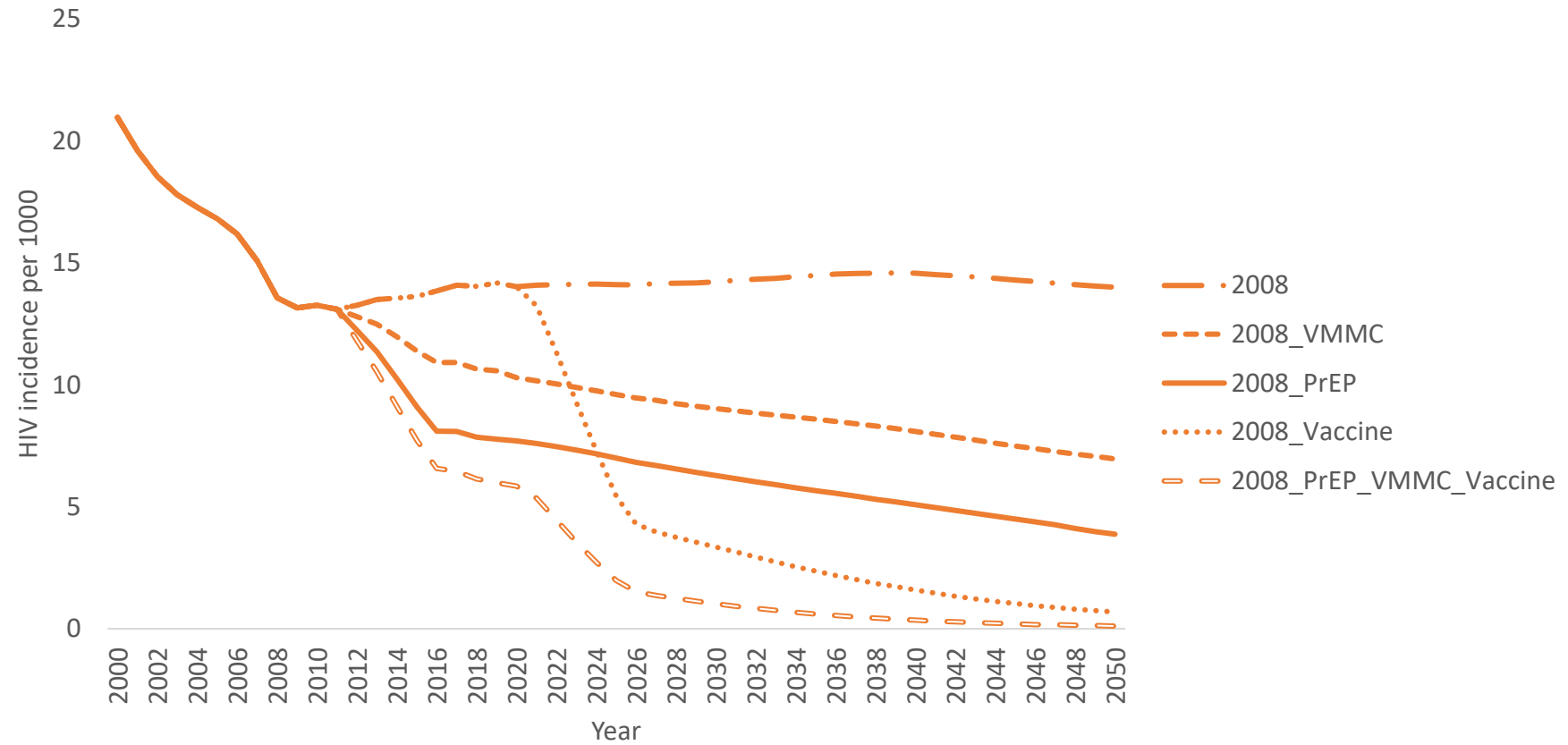

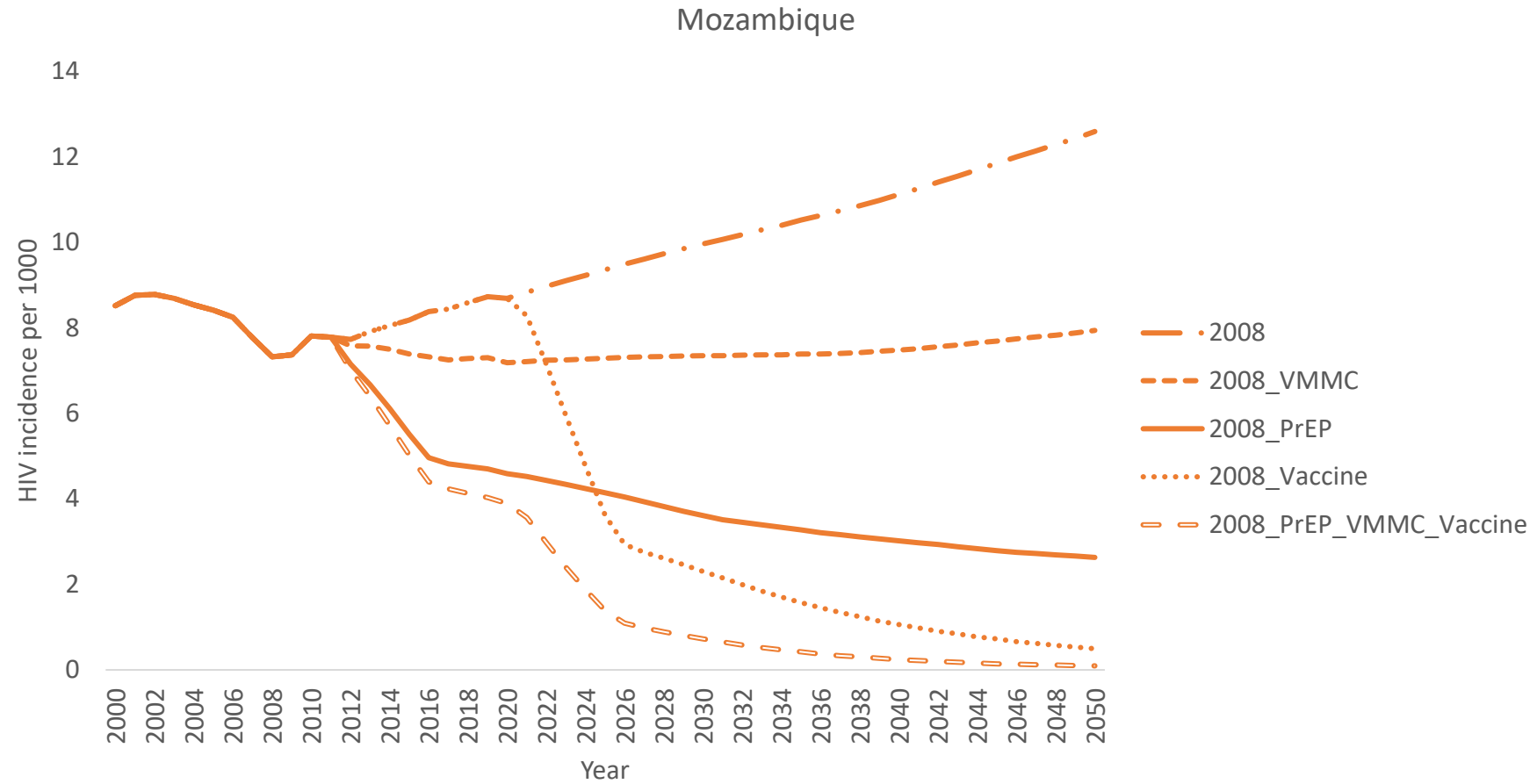

## Uganda

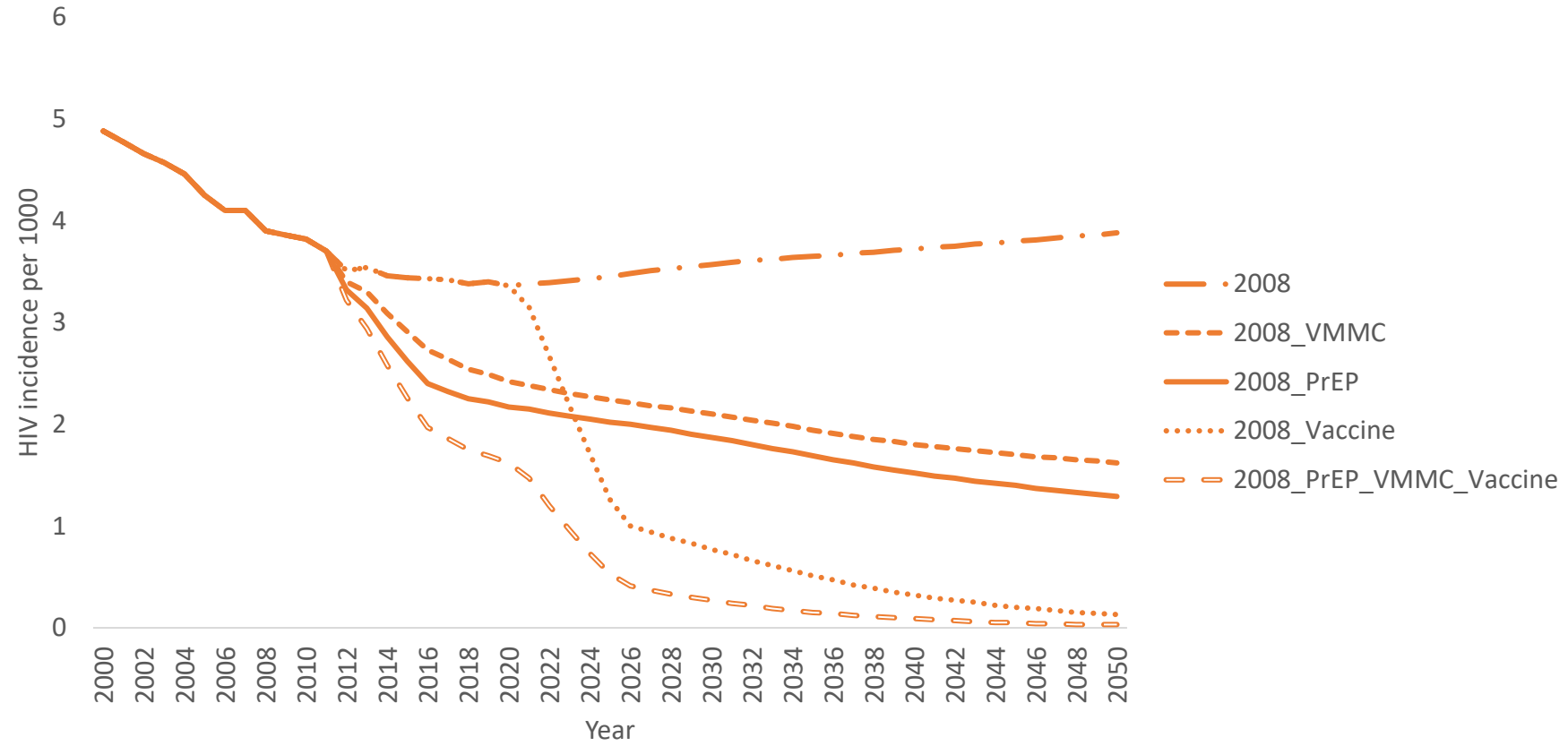

Supplement: Supplementary file 2 [file mmc2.pdf]

## Lesotho

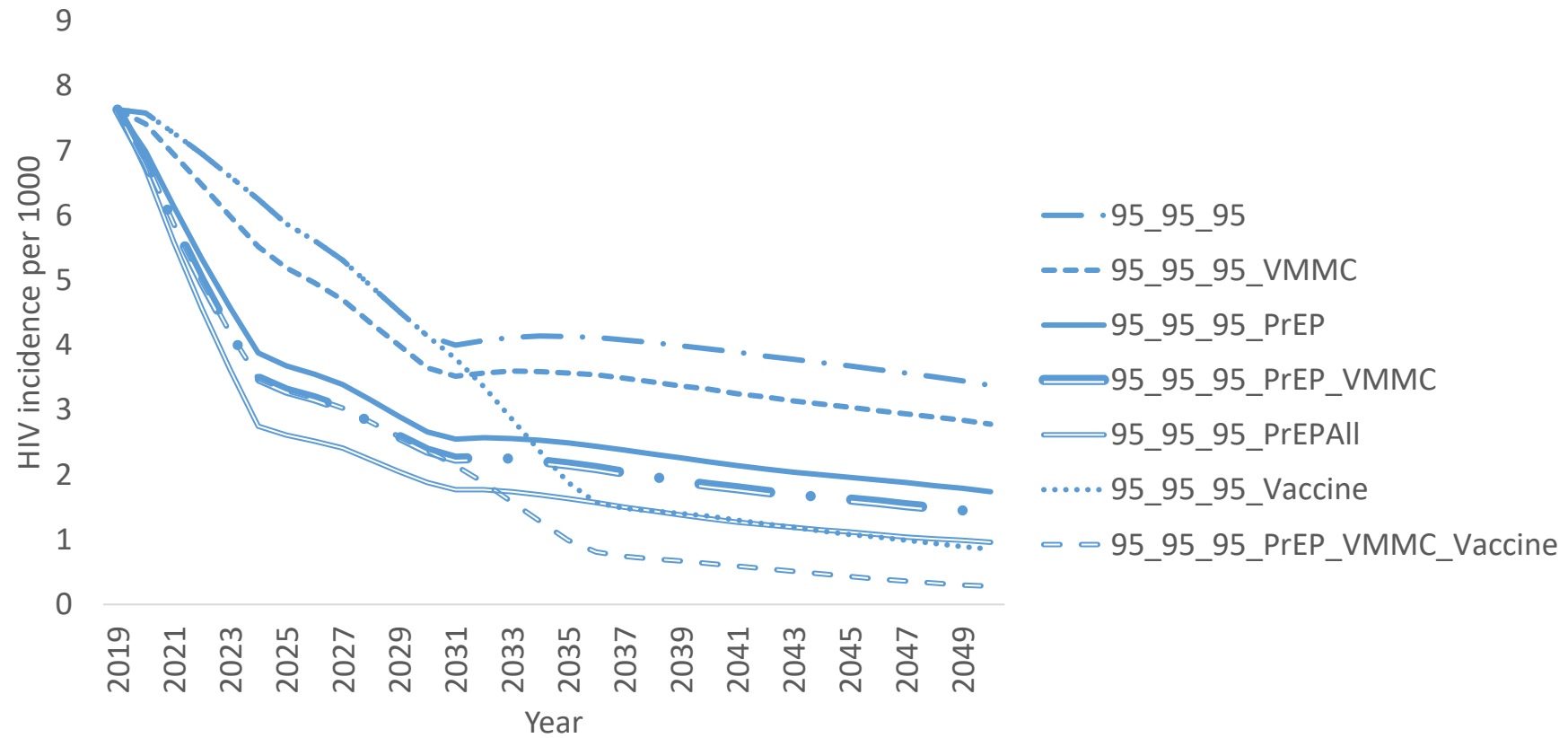

Mozambique

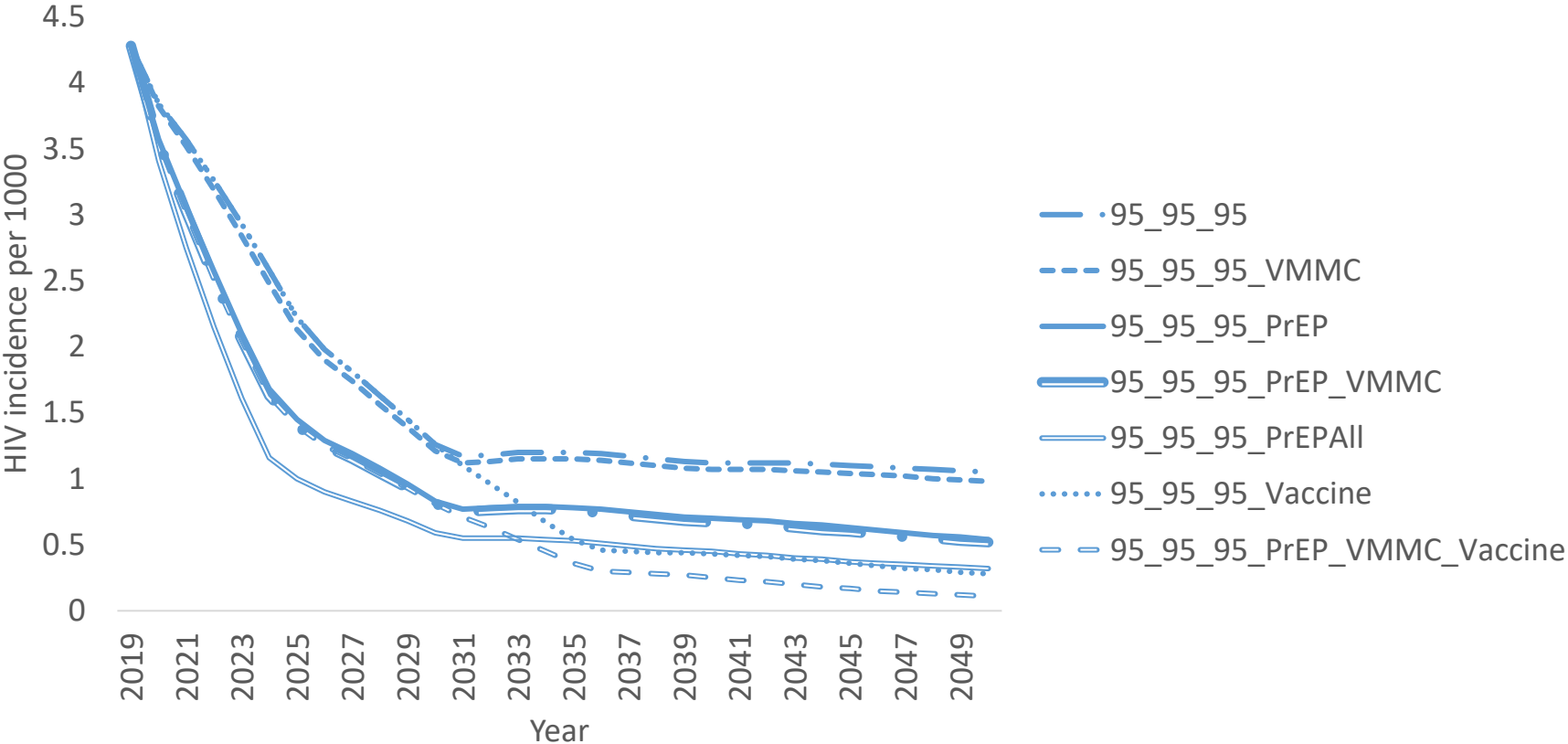

## Uganda

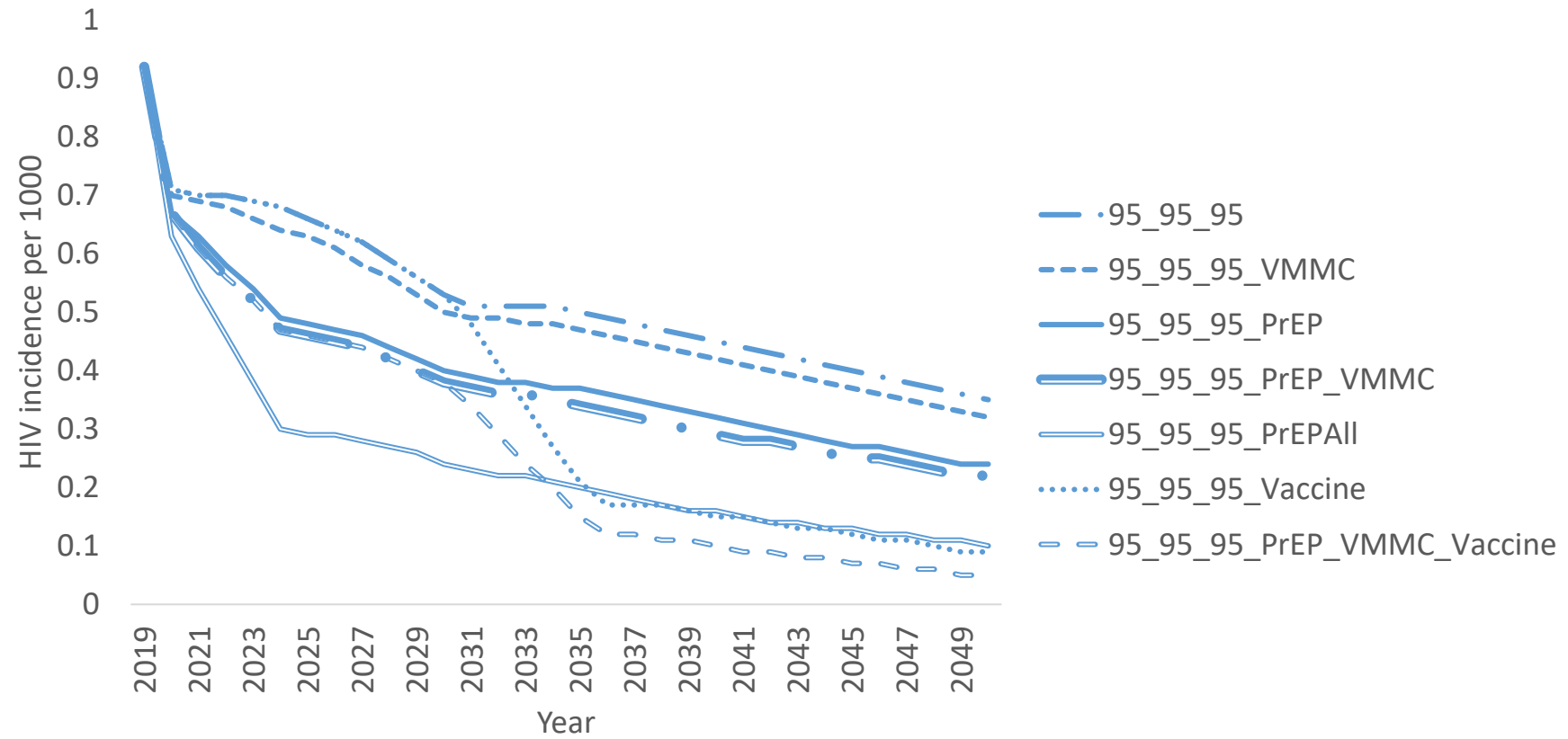

Supplement: Supplementary file 3 [file mmc3.pdf]

## Lesotho

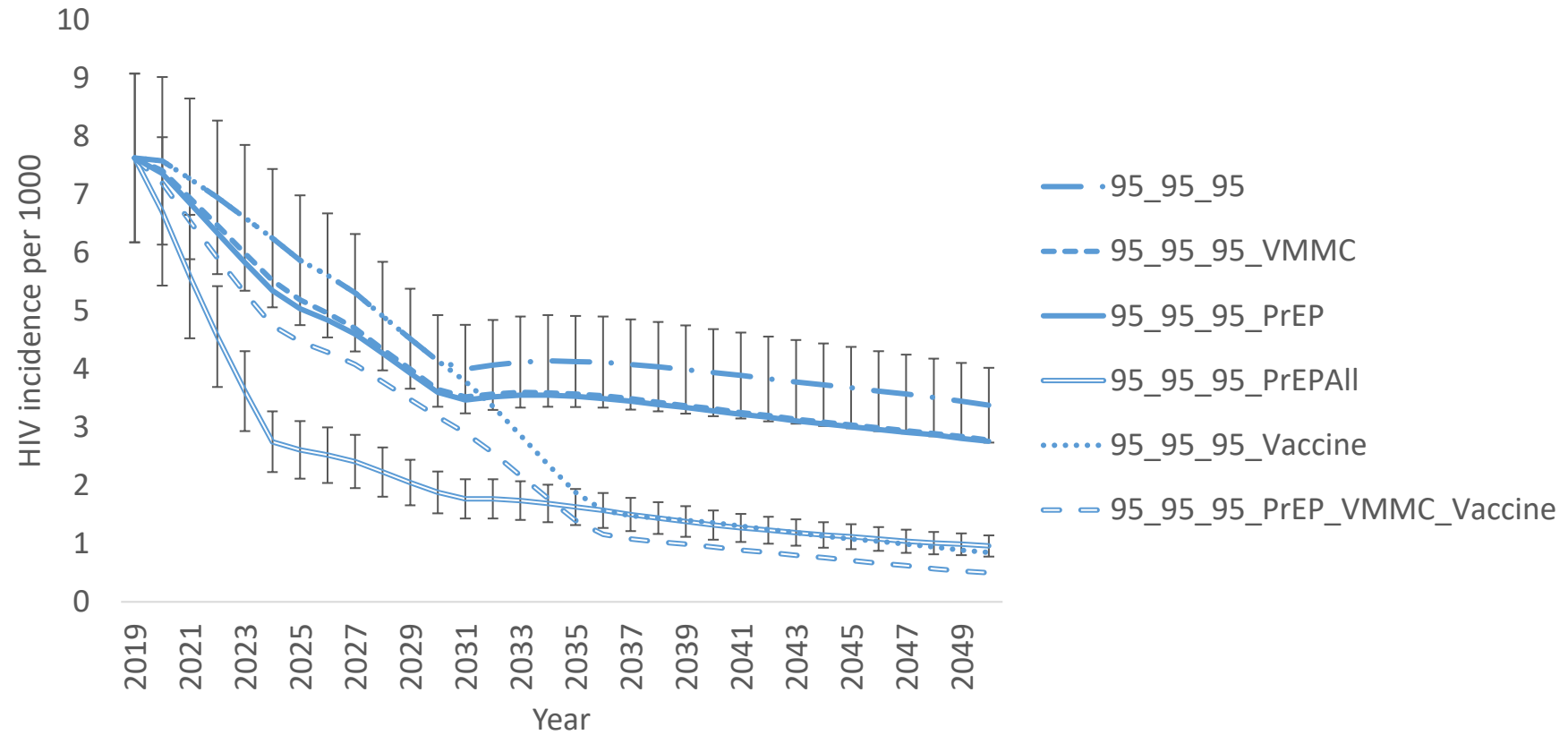

Mozambique

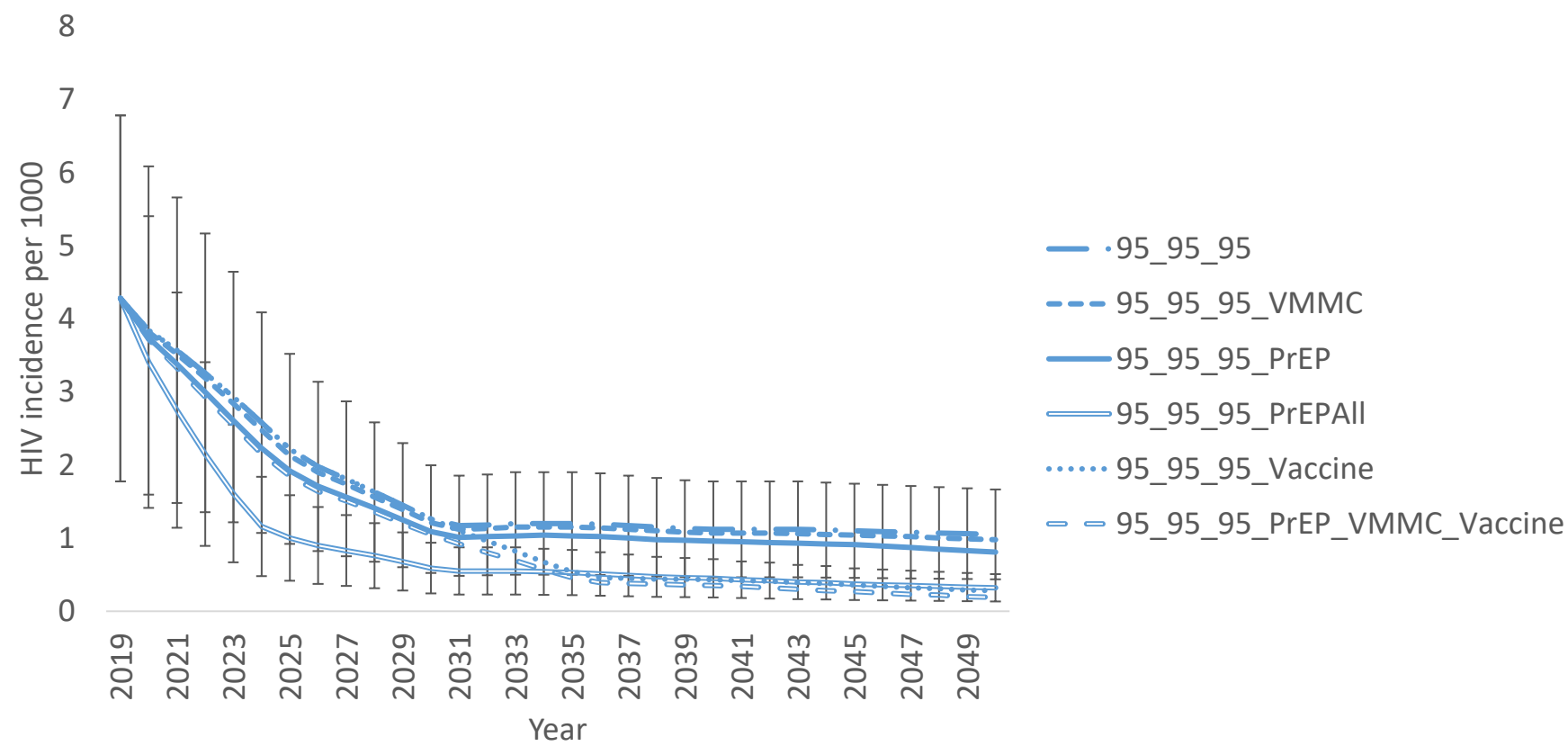

## Uganda

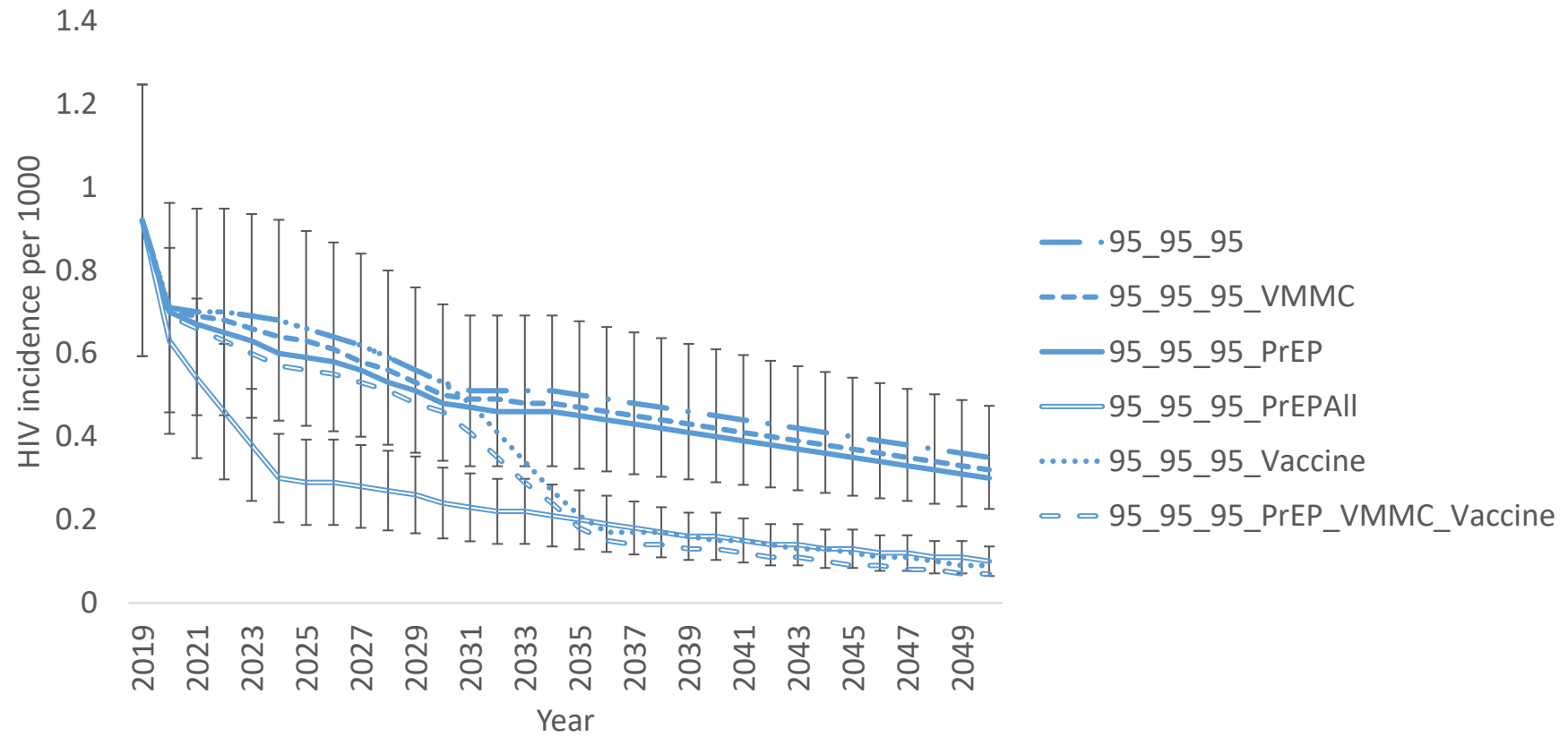

Supplement: Supplementary file 4 [file mmc4.pdf]
